# Too Jittery to Sleep? Temporal Associations of Actigraphic Sleep and Caffeine in Adolescents

**DOI:** 10.3390/nu14010031

**Published:** 2021-12-23

**Authors:** Gina Marie Mathew, David A. Reichenberger, Lindsay Master, Orfeu M. Buxton, Anne-Marie Chang, Lauren Hale

**Affiliations:** 1 Program in Public Health, Department of Family, Population, and Preventive Medicine, Renaissance School of Medicine, Stony Brook University, Stony Brook, NY 11794, USA; lauren.hale@stonybrookmedicine.edu; 2 Department of Biobehavioral Health, College of Health and Human Development, The Pennsylvania State University, University Park, PA 16802, USA; reichenberger@psu.edu (D.A.R.); lmm5346@psu.edu (L.M.); orfeu@psu.edu (O.M.B.); amchang@psu.edu (A.-M.C.)

**Keywords:** sleep duration, sleep timing, sleep maintenance efficiency, subjective sleep quality, sleep variability, social jetlag, caffeine, adolescence, actigraphy, diary

## Abstract

Caffeine consumption has been linked to poor sleep health in adolescents, but it is unknown whether poor sleep predicts caffeine consumption, and/or whether caffeine consumption predicts poor sleep, particularly when sleep is measured objectively. Data were collected from a micro-longitudinal sub-study of the age 15 wave of the Fragile Families and Child Wellbeing Study (*n* = 589). Adolescents wore an actigraphy device and completed daily surveys for ~1 week. Daily surveys assessed subjective sleep quality and caffeinated beverage consumption (0 = no caffeine, 1 = any caffeine). Separate mixed models assessed whether actigraphy-measured sleep duration, timing, maintenance efficiency, and subjective quality predicted next-day caffeinated beverage consumption within and between adolescents. Variability (standard deviation) of sleep duration and timing, sleep regularity index, and social jetlag were tested as additional between-person predictors. Lagged models tested whether daily caffeinated beverage consumption predicted sleep that night (*n* = 458). Adolescents with more variable sleep duration and midpoint had higher average odds of consuming caffeinated beverages compared to others. After adolescents consumed ≥1 caffeinated beverage, they had later sleep onset that night and wake time the next morning than usual versus when they did not consume caffeine. Curbing caffeinated beverage consumption may aid in the maintenance of regular sleep schedules and advance sleep timing in adolescents.

## 1. Introduction

Caffeine is a stimulant drug readily available in the United States and present in coffee, black and green tea, some sodas, and energy drinks [1]. Consumption of caffeine is high among American adolescents, with 75% reporting consuming a caffeinated beverage on a typical day [2,3]. Adolescents who consume caffeine may become caffeine dependent [4], and high caffeine consumption is associated with cigarette use, psychosocial problems [5], and lower grade point average [6] in adolescents. The high prevalence of regular caffeine consumption in adolescents and the potential negative consequences on health and wellbeing indicate the need for more research into the antecedents and consequences of caffeine consumption in this population.

The potential negative effects of adolescent caffeine consumption include short sleep duration [2,6,7,8,9,10,11,12,13,14,15,16,17,18,19]. Over 70% of American high schoolers report obtaining fewer than the recommended minimum of 8 h per night [20]. Cross-sectional studies measuring sleep through self-report generally find that adolescents who consume more caffeine have shorter sleep duration [2,6,7,8,9,10,11,12,13,14,15,16,17,18,19]. Some studies with similar designs, however, have found no association between caffeine and sleep duration in adolescents [21,22,23]. The few studies examining the link between objectively measured sleep (i.e., through wrist actigraphy) and caffeine consumption in adolescents have also demonstrated no association [24,25], warranting further research with objective measures of sleep.

Later sleep timing has also been linked to caffeine consumption in adolescents [6,8,12,22,23,24,26,27,28,29,30]. Adolescents tend to have a later chronotype, or preference for timing of sleep and other behaviors, than children or adults [31]. Studies employing a cross-sectional design and measuring sleep through self-report have found that greater caffeine consumption is associated with later sleep timing [6,8,12,22,23,24,27,28] and evening preference [26,29,30] in adolescents, with one finding no association between caffeine consumption and evening preference [32]. There is a lack of research on the association between caffeine consumption and objectively measured sleep timing in adolescents.

Few studies have examined the association between caffeine consumption and other dimensions of sleep, such as quality (whether measured objectively or through self-report) and variability, in adolescents. One study demonstrated that higher caffeine intake was associated with greater self-reported wake after sleep onset (a measure of poor sleep quality) [19], and adolescents who consumed at least two servings of caffeine per day demonstrated less slow-wave activity (indicating poorer sleep quality) as measured through polysomnography (PSG), the gold standard for sleep monitoring [33], compared to adolescents who did not consume caffeine [24]. However, two studies that measured sleep through actigraphy found no association between caffeine consumption and wake after sleep onset in adolescents [24,25], and another found a null association between caffeine consumption and self-reported sleep quality [23]. These mixed findings indicate the need for more research on the associations of objective and subjective sleep quality with caffeine consumption in adolescents. There is also a lack of research on the associations of variability in sleep duration and timing, important dimensions of sleep that suggest poorer sleep health [34], with caffeine consumption in adolescents.

The association between sleep and caffeine may be bidirectional. Some adolescents report consuming caffeinated beverages to counteract daytime sleepiness and fatigue [35], and caffeine consumption may reduce that night’s sleep duration and quality [36]. However, most studies that examined the association between sleep and caffeine consumption in adolescents employed a cross-sectional design [2,5,6,7,8,9,10,11,12,13,14,15,16,18,20,21,22,23,24,25,26,27,28,29,31], which precludes examination of temporal associations. Whereas cross-sectional studies are only able to describe between-person associations [37] (e.g., whether adolescents who sleep longer on average tend to consume caffeine more often than other adolescents), longitudinal studies can establish temporal precedence [38] (e.g., whether adolescents are more likely to consume caffeine the next morning following nights when they sleep shorter than usual). Only a few studies have examined longitudinal associations between caffeine consumption and dimensions of sleep among adolescents. Two studies found no within-person associations when measuring sleep through self-report [18,39]. One micro-longitudinal study measuring sleep through PSG found that increased caffeine consumption predicted less total sleep time and sleep efficiency that night, and reduced sleep efficiency predicted more afternoon caffeine consumption the next day [40], but the study did not assess sleep timing, subjective quality, or variability and included fewer than 100 adolescents. Determining the direction of the sleep—caffeine relationship while assessing multiple dimensions of sleep may allow for future targeted interventions to improve health behaviors, including sleep and dietary habits.

There is minimal research examining temporal within-person associations between caffeine consumption and objectively measured sleep among adolescents. Self-reported sleep can deviate considerably from sleep assessed through objective measures such as actigraphy [41], and between-person effects assessed in cross-sectional research do not necessarily translate to within-person effects in direction or magnitude [37]. It may be particularly important to examine associations between caffeine consumption and dimensions of sleep health given that adolescents begin to gain autonomy over their own sleep behaviors [42] and diet [43] versus childhood.

The present study examined bidirectional associations of objectively measured sleep dimensions and subjective sleep quality with caffeinated beverage consumption in adolescents. We additionally examined whether sleep variability was associated with greater caffeinated beverage consumption between adolescents. We hypothesized that poor sleep health (shorter or longer sleep duration, later sleep timing, lower sleep maintenance efficiency, and lower self-reported sleep quality) would predict caffeinated beverage consumption the next day, and that caffeinated beverage consumption would predict poor sleep health that night. We further hypothesized that greater variability in sleep duration and timing would be associated with more caffeinated beverage consumption between adolescents.

## 2. Materials and Methods

### 2.1. Participants

Data for the current analyses come from the Fragile Families and Child Wellbeing Study (FFCWS; www.fragilefamilies.princeton.edu, accessed on 11 August 2020), a longitudinal birth cohort oversampled for nonmarital births, which resulted in a greater proportion of racial/ethnic minority mothers and those of lower socioeconomic status and education level compared to the national population. More details regarding the sample and design may be found elsewhere [44]. Survey data from the Fragile Families and Child Wellbeing study (https://fragilefamilies.princeton.edu/documentation, accessed on 22 March 2017) are publicly available from Princeton University’s Office of Population Research (OPR) data archive: https://opr.princeton.edu/archive/restricted/Default.aspx, accessed on 22 March 2017. The sleep actigraphy and daily diary datasets generated and analyzed during the current study are not publicly available yet, but will be available through an application process at the above link. This study was conducted according to the guidelines laid down in the Declaration of Helsinki of 1975 (revised 2013), and all procedures involving human participants were approved by the Princeton University and Stony Brook University (CORIHS B) (FWA #00000125) Institutional Review Boards. Written (for in-home interviews) or recorded verbal (for phone interviews) informed consent was obtained from primary caregivers, and assent was obtained from adolescents.

The original FFCWS birth cohort consists of 4898 children born from 1998–2000 in 20 large cities in the United States [45]. Families were recruited from local hospitals at the time of the child’s birth. The study staff maintained records about the participants and their families for follow up at subsequent waves, when participants were approximately ages 1, 3, 5, 9, and 15 years of age. Families were eligible for inclusion in the age 15 follow-up wave if the child was alive and not legally adopted (95% of the birth sample were eligible). Data in the current analyses were collected from February 2014 to March 2016. During the year 15 wave of the FFCWS (wave 6), 3444 adolescents and their primary caregivers completed separate surveys querying household and demographic characteristics, administered either over the phone or in person at the participant’s place of residence. A randomly selected subsample (*n* = 1090) was asked to participate in a micro-longitudinal FFCWS sub-study. Adolescents who agreed to participate (*n* = 1049) were asked to wear a wrist-worn accelerometer at all times and answer a daily diary for seven consecutive days in the evening.

The design of the study was such that a night’s sleep predicted the next day’s diary report, including assessment of caffeinated beverage consumption. Therefore, lagging analyses to examine if caffeinated beverage consumption predicted that night’s sleep required dropping the first sleep recording and the last diary report. In the originally structured non-lagged dataset (where nighttime sleep predicted next-day caffeinated beverage consumption), of *n* = 1049 assenting adolescents, *n* = 414 were excluded due to not providing at least 3 valid days of actigraphy recordings (dataset has average of 5.6 ± 1.4 recordings per adolescent; range 3–10; IQR 5–7) and next-day diary reports (dataset has average of 5.5 ± 1.4 reports per adolescent; range 3–9; IQR 4–7), and *n* = 46 were excluded due to not reporting whether they consumed a caffeinated beverage on at least three days, leaving a total sample of *n* = 589 adolescents (56.1% of the subsample). A further *n* = 219 adolescents were excluded from social jetlag analyses due to not providing at least one school night and one non-school night, resulting in *n* = 370 included adolescents.

For analysis of caffeinated beverage consumption predicting that night’s sleep (in the lagged dataset), of *n* = 1049 assenting adolescents, *n* = 492 adolescents were excluded due to not providing at least 3 valid lagged days of actigraphy recordings (lagged dataset has average of 5.2 ± 1.1 recordings per adolescent; range 3–9; IQR 4–6) and next-day diary reports (lagged dataset has average of 4.7 ± 1.2 reports per adolescent; range 3–8; IQR 4–6), *n* = 98 adolescents were excluded due to not reporting whether they consumed a caffeinated beverage on at least three lagged days, and *n* = 1 adolescent was excluded due to missing body mass index data, leaving *n* = 458 adolescents included in the lagged analyses (43.7% of the subsample). Supplemental Figure S1 (for sleep predicting caffeinated beverage consumption) and S2 (for caffeinated beverage consumption predicting sleep in lagged analyses) depict participant flow charts, and a “Strengthening the Reporting of Observational Studies in Epidemiology—Nutritional Epidemiology” (STROBE-nut) checklist is included as Supplemental Table S1 [46].

Separate logistic regression analyses were conducted to compare sex, race/ethnicity, and income between the adolescents included in the analyses and those who assented to participate in the sub-study but were ultimately excluded due to missing data (assented *n* = 1049). In non-lagged analyses (where sleep predicted next-day caffeinated beverage consumption; analytical sample *n* = 589, excluded *n* = 460), male sex marginally predicted missingness (odds ratio, OR = 1.13, *p* = 0.052), and Black/African American race/ethnicity (vs. White/Caucasian; OR = 1.38, *p* < 0.001) and lower household income (in thousands of dollars; OR = 0.997, *p* = 0.012) predicted higher odds of data missingness. In lagged analyses (where caffeinated beverage consumption predicted that night’s sleep; analytical sample *n* = 458, excluded *n* = 591), Black/African American race/ethnicity (OR = 1.31, *p* = 0.005) and lower household income (OR = 0.998, *p* = 0.020) predicted higher odds of data missingness. All analyses were adjusted for sex, race/ethnicity, and household income.

### 2.2. Actigraphy Device and Scoring

Sleep measures were collected with a wrist-worn accelerometer with off-wrist detection (Actiwatch Spectrum; Philips-Respironics, Murrysville, PA, USA) and study participants were asked to wear the watch on their non-dominant wrist for one week. Data from the Spectrum device were downloaded with Actiware software (Version 6.0.4, Philips-Respironics, 2017). At least two trained independent scorers (blinded to each other) scored the data using an algorithm validated against polysomnography [33]. Sleep diaries were not used for actigraphy scoring given the frequent inaccuracy, uncertainty, and potential for systematic bias caused by discrepant diary and actigraphy reports [47]. The two scorers looked for disagreements in the number of valid days, cut-time (i.e., start and end time that defines a 24 h day), presence of all-nighters (i.e., no sleep interval within a 24 h day), the presence of false on-wrist detection, presence of naps, number of sleep intervals, and each sleep onset or offset that differed by >15 min. The scorers met to resolve any discrepancies related to these aspects of the data, and a final dataset was established [48]. The scorers determined sleep intervals using a decrease in activity levels and the aid of light levels for sleep onset and sleep offset [49], and a nighttime sleep interval was split into two intervals (main sleep and nap) if there was an awakening ≥1 h during this interval. A sleep actigraphy day was determined invalid and no sleep interval was set if there were ≥4 total hours of off-wrist time, with the exception of the first and last day (device should have been worn at least 2 h on the first day). Other invalidation criteria were constant false activity due to battery failure, data unable to be retrieved or recovered, or an off-wrist period of ≥60 min within 10 min of the scored beginning or end of the main sleep period for that day.

### 2.3. Daily Alignment of Sleep and Diary and Data Lagging

Participants were asked to complete a diary each evening after 7:00 PM (19:00) and before going to sleep. Sleep and daily diary data were merged by participant identification number and date/time. A previous night’s sleep and the following day’s diary answers were aligned on the same observation record in the originally structured merged data. Nights were excluded from analyses if the adolescent had an all-nighter and received no sleep.

To investigate whether caffeinated beverage consumption predicted that night’s sleep, diary entries were each lagged by one row (i.e., day) through the “lag1” function in SAS 9.4. Lagging data resulted in one row of data loss per adolescent and reduced the sample size, due to adolescents requiring 4 non-lagged days to produce 3 lagged days.

### 2.4. Variables

#### 2.4.1. Nightly Actigraphic Sleep Measures

Sleep onset and sleep offset were the start and end of the main nighttime sleep interval calculated in hours from midnight, respectively. Sleep midpoint (also midnight-centered) was calculated as midway between sleep and sleep offset. Sleep duration was calculated as the number of hours between sleep onset and sleep offset of the main nighttime sleep interval. Nighttime sleep maintenance efficiency (considered an objective measure of sleep quality) represents the percent of sleep duration that the individual spent asleep and was calculated as 1 − (wake after sleep onset in hours/sleep duration in hours) and multiplied by 100 to produce a percentage [50].

#### 2.4.2. Actigraphic Sleep Measures Calculated per Adolescent

We calculated measures of sleep variability per adolescent across the monitoring days in the non-lagged dataset (*n* = 589). Sleep duration variability, sleep onset variability, sleep midpoint variability, and sleep offset variability were calculated as the standard deviation (*SD*, per adolescent) of each measure. Sleep regularity index (SRI) was calculated based on the formula from Phillips et al. [51] and ranges from 0 (low regularity) to 100 (high regularity). The score represents the percentage probability that an individual is in the same state (sleeping or awake) at any two time points 24 h apart, averaged across the interval of measurement. Social jetlag, a misalignment of sleep midpoint between school and free days, was calculated in hours through the following formula: |sleep midpoint on free nights−sleep midpoint on school nights| [52]. Only adolescents with at least one school night and one free night were included in the social jetlag measure; therefore, some adolescents did not have social jetlag values (*n* = 370 of the 589 total adolescents had social jetlag values).

#### 2.4.3. Subjective Sleep Quality

Adolescents rated their last-night sleep quality on the daily diary by answering, “How would you rate your sleep quality?” with possible answers of “very good” (1), “fairly good” (2) “fairly bad” (3) or “very bad” (4). This variable was reverse coded such that “very bad” became 0, and “very good” became 3.

### 2.5. Caffeinated Beverage Consumption

Adolescents were asked on the daily diary, “How many caffeinated beverages (such as coffee, soda, energy drinks) did you have? One beverage is about 8 ounces” with response options of 0 (0), 1 (1), 2 (2), 3 (3), 4 (4), or 5 or more (5). Items were “coffee or tea (iced or hot),” “caffeinated soda (such as Coca-cola, Pepsi, Mountain Dew),” and “energy drinks (such as Red Bull, Monster, 5-h Energy, RockStar, Full Throttle, Amp, etc.).” A “number of caffeinated beverages that day” variable was constructed as the sum of coffee/tea, caffeinated soda, and energy drinks consumed each day. Due to the considerable number of days on which adolescents reported not consuming any caffeinated beverages (39.7% of the 3215 observations), the caffeinated beverages variable was dichotomized into 0 = none; 1 = at least one 8 oz beverage that day.

### 2.6. Covariates

Adolescents also reported whether they went to school (0 = no school, 1 = school) on the daily diary.

Other covariates were assessed through surveys administered once to youth and their primary caregivers during the year 15 wave. Race/ethnicity was reported on the youth survey and grouped into exclusive categories of White/Caucasian (not Hispanic or Latino), Black/African (not Hispanic or Latino), Hispanic and/or Latino (any race), or a category with other (including Asian, Central American/Caribbean, Native American/Alaska Native, and/or Native Hawaiian/Pacific Islander), mixed, and no race/ethnicity reported. The primary caregiver’s education level (“some high school,” “completed high school,” “some college,” or “college graduate”), annual income (in USD), and whether the youth lived with two biological parents were assessed on a survey administered to the youth’s primary caregiver.

Information about biological sex was collected at birth. Weight and height were assessed objectively during in-person interviews by trained research assistants. Body mass index (BMI) percentile (range 0–100) was calculated based on the 2000 Centers for Disease Control and Prevention (CDC) growth charts [53], which matches BMI (weight in kg/(height in m^2^)) [54] for the adolescent’s sex and age.

### 2.7. Statistical Analyses

Analyses were conducted in SAS 9.4 software (SAS Institute, Cary, NC, USA). Most variables met standards for normality (skew < |3| and kurtosis < |10|) [55]. Variability (*SD*s) of sleep onset, midpoint, and offset and social jetlag were positively skewed (skew ≥ 3) and/or leptokurtotic (kurtosis ≥ 10) and were winsorized (i.e., values beyond the 99th percentile were replaced with the 99th percentile value). Of 589 adolescents, 5 sleep onset *SD* values, 5 sleep midpoint *SD* values, 6 sleep offset *SD* values, and 2 social jetlag values were winsorized. After winsorization, these variables also met criteria for normality.

Within-person reliability for each repeated measure (i.e., assessed through actigraphy or diary) was analyzed. Intraclass correlation coefficients (ICCs) were obtained for continuous measures (random effect variance/total variance [56]), which included all nightly sleep measures and number of caffeinated beverages. Pseudo-ICCs were obtained for binary measures (generated by the generalized estimating equation procedure [57,58]), which included the dichotomized caffeinated beverage consumption and school attendance. Higher ICCs indicate less variation within adolescents.

#### 2.7.1. Main Analyses

In non-lagged analyses where sleep predicted next-day caffeinated beverage consumption, multilevel models (PROC GLIMMIX, with a binary distribution for the outcome caffeine consumption) tested whether dimensions of nightly sleep (sleep duration, sleep onset, midpoint, and offset, and sleep maintenance efficiency, measured through actigraphy; subjective sleep quality, measured through diary) predicted the odds of consuming ≥1 caffeinated beverage the next day, within adolescents. A quadratic association between sleep duration and odds of caffeinated beverage consumption was assessed using the predictor sleep duration^2^ (sleep duration × sleep duration). The between-person predictor (within the same model as the within-person predictor) tested whether average sleep per adolescent across the monitoring period was associated with average odds of consuming ≥1 caffeinated beverage.

For the sleep measures that varied within-person (sleep duration, onset, midpoint, offset, maintenance efficiency, and subjective sleep quality), two-level models examined 3215 total daily observations that were clustered within 589 adolescents. Variances for nightly sleep measures were decomposed into within-person (level-1) and between-person (level-2) levels [38]. Within-person predictor variables were centered around the person mean, such that positive values indicated that value was higher than the person’s own cross-day average. Between-person predictor variables were calculated as the mean per person across all time points. We specified the denominator degrees of freedom to be computed by dividing the residual degrees of freedom into between-subject and within-subject portions (DDFM = BETWITHIN) [59]. For the sleep variability measures that were between-person only (*SD* of duration, onset, midpoint, and offset, SRI, and social jetlag), the analyses were conducted with the sleep variability measure predicting the odds of consuming ≥1 caffeinated beverage (on average) per adolescent. Covariates that were significantly associated with odds of caffeinated beverage consumption were also included in models: school attendance and primary caregiver’s highest education level. Analyses with predictors other than sleep duration were further adjusted for sleep duration and sleep duration^2^.

Lagged analyses with caffeinated beverage consumption predicting sleep that night were conducted similarly as analyses that predicted next-day caffeinated beverage consumption from sleep, except using PROC MIXED for the continuous sleep outcomes. There were 2128 observations nested within 458 adolescents. Covariates that were significantly associated with any sleep outcome were also included in models: school attendance, BMI percentile, primary caregiver’s education level, and whether the adolescent was living with two biological parents.

All models used autoregressive (AR) (1) covariance structure, included a random intercept for participant variation, and adjusted for sex, race/ethnicity, and household income. Alpha < 0.05 (two-sided) was deemed statistically significant.

#### 2.7.2. Sensitivity Analyses by Adolescent’s Average Caffeinated Beverage Consumption

To examine whether the associations between sleep and caffeinated beverage consumption varied as a function of the adolescent’s average consumption, we conducted two sets of sensitivity analyses. One set of analyses examined whether the within-person effect of sleep on next-day caffeinated beverage consumption varied as a function of the proportion of days each adolescent consumed caffeine (a between-person moderator). These analyses included the interaction term within-person dimension of sleep x proportion of days the adolescent consumed caffeine predicting next-day caffeinated beverage consumption. The second set of analyses examined whether the within-person effect of caffeine on that night’s sleep varied as a function of the proportion of days each adolescent consumed caffeine (lagged analyses). These analyses included the interaction term within-person caffeinated beverage consumption × proportion of days the adolescent consumed caffeine predicting sleep that night.

## 3. Results

### 3.1. Demographic Information

For the non-lagged analyses where sleep predicted caffeinated beverage consumption, 589 adolescents provided at least 3 nights of actigraphy, next-day diary, and complete covariate data (53% female, *n* = 311; mean age ± *SD* = 15.4 ± 0.5 years, range 14.7–17.7), with an average of 5.6 ± 1.4 actigraphy nights per adolescent (range 3–10 days; IQR 5–7) and 5.5 ± 1.4 reports of caffeinated beverage consumption (range 3–9 days; IQR 4–7). Ethno-racial composition of the sample was as follows: 41% Black/African American (*n* = 240), 25% Hispanic or Latino (*n* = 149), 19% White/Caucasian (*n* = 112), and 15% other, mixed, or none (*n* = 88). The mean percent of days that adolescents reported consuming ≥1 caffeinated beverage was 61% ± 34%. Demographic information and caffeinated beverage consumption were similar for lagged analyses (where caffeinated beverage consumption predicted sleep, *n* = 458 adolescents); the average number of actigraphy nights provided by each adolescent was 5.2 ± 1.1 (range 3–9 days; IQR 4–6) and the average number of reports of caffeinated beverage consumption was 4.7 ± 1.2 (range 3–8; IQR 4–6). Other sample information for the non-lagged dataset (*n* = 589), including descriptive statistics for sleep variables of interest and covariates, is in Table 1.

The ICCs for sleep measures ranged from 0.15 to 0.53, indicating poor to moderate reliability and therefore considerable within-person variation [56]. The ICC for the continuous caffeinated beverages variable (number of caffeinated beverages on a given day) was 0.58, and the pseudo-ICC for the dichotomized caffeinated beverages variable (caffeinated beverage consumption) was 0.39, demonstrating that the dichotomized measure had higher within-person variability. ICC and pseudo-ICC values for each nightly sleep measure, caffeinated beverages (continuous and dichotomized), and school attendance are in Supplemental Table S2.

### 3.2. Nightly Sleep Measures Predicting Next-Day and Average Caffeinated Beverage Consumption (Within- and Between-Person Associations)

No dimension of sleep measured nightly (sleep duration, onset, midpoint, offset, maintenance efficiency, or subjective quality) predicted next-day caffeinated beverage consumption within adolescents, nor were there any significant between-person associations for any of these sleep measures with odds of consuming ≥1 caffeinated beverage (all *p* ≥ 0.10).

We additionally examined whether the within-person effects of sleep on next-day caffeinated beverage consumption varied depending on adolescent’s average caffeinated beverage consumption (i.e., the proportion of days on which an adolescent reported consuming ≥1 caffeinated beverage). There were no significant interactions (all *p* > 0.26).

### 3.3. Associations of Sleep Variability with Average Caffeinated Beverage Consumption (Between-Person Associations)

**Figure 1:**
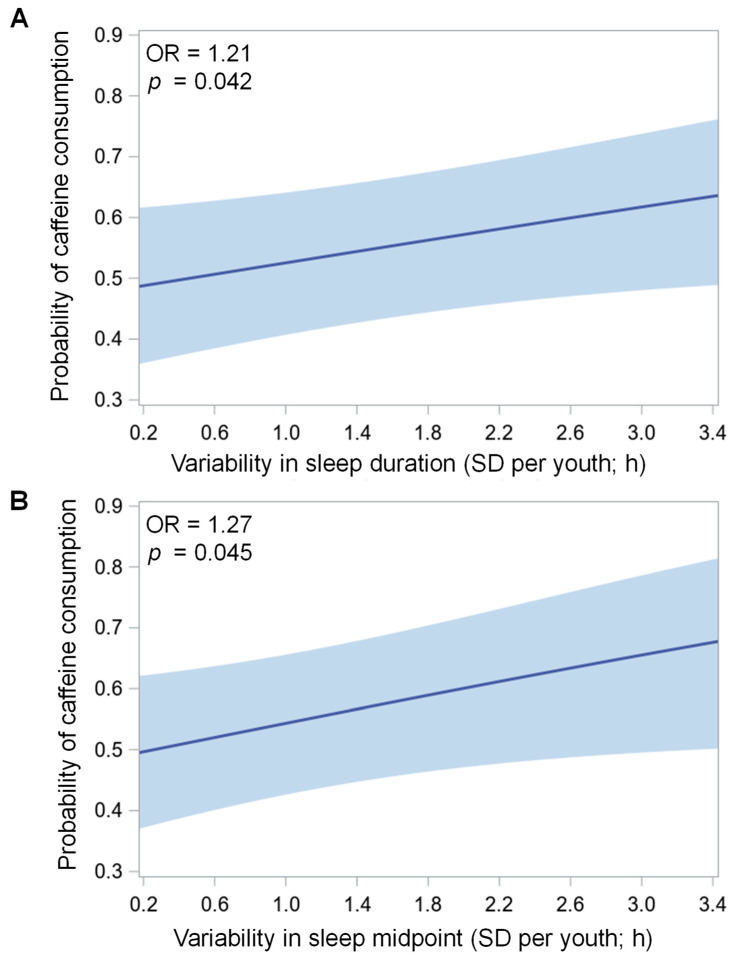
Associations of variability in sleep duration (**A**) and midpoint (**B**) (each in standard deviation, *SD* hours) per youth with average probability of caffeinated beverage consumption (0 = none; 1 = at least one 8 oz beverage that day), which includes coffee or tea, caffeinated soda, and energy drinks, across monitoring days in two separate mixed models. The mean number of valid actigraphy nights per youth included in present analyses was 5.6 ± 1.4 (range 3–10; interquartile range, IQR 5–7) and the mean number of reports of caffeinated beverage consumption was 5.5 ± 1.4 (range 3–9; IQR 4–7). Both models adjust for mean sleep duration (linear and quadratic, sleep duration x sleep duration) and demographic/household covariates: birth sex, race/ethnicity, household income, and primary caregiver’s education level. Shaded light blue bands depict 95% confidence interval of mean probability of caffeinated beverage consumption per youth predicted from each sleep measure. H, hours; OR, odds ratio; SD, standard deviation.

There were significant associations between variability of sleep duration (*p* = 0.042) and variability of sleep midpoint (*p* = 0.045) with average odds of caffeinated beverage consumption, such that for every one *SD*-hour increase in variability, the average odds of an adolescent consuming one or more caffeinated beverages on a given day increased by 21% and 27%, respectively (see Figure 1 and Table 2). Variability of sleep onset (*p* = 0.093) and variability of sleep offset (*p* = 0.058) were marginally associated with average odds of caffeinated beverage consumption, such that for every one *SD*-hour increase in variability, the average odds of an adolescent consuming one or more caffeinated beverages on a given day increased by 19% and 17%, respectively. There were no significant associations of SRI (*p* = 0.214) or social jetlag (*p* = 0.420) with caffeinated beverage consumption.

### 3.4. Caffeinated Beverage Consumption Predicting Sleep Measures That Night and on Average (Within- and Between-Person Associations)

**Figure 2:**
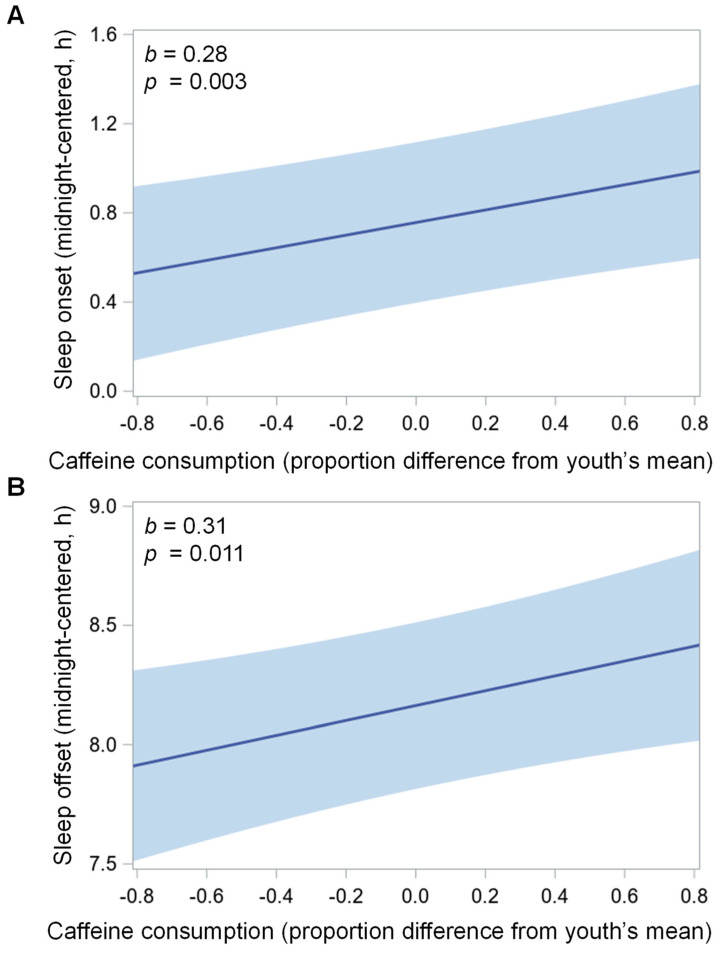
Caffeinated beverage consumption (0 = none; 1 = at least one 8 oz beverage that day), which includes coffee or tea, caffeinated soda, and energy drinks, predicting sleep (**A**) onset and (**B**) offset (each in hours from midnight) that night within each adolescent in two separate mixed models. Positive *x*-axis values indicate the adolescent consumed ≥1 caffeinated beverage that day; negative values indicate the adolescent did not consume a caffeinated beverage that day. The mean number of valid actigraphy nights per youth included in present analyses was 5.2 ± 1.1 (range 3–9; interquartile range, IQR 4–6) and the mean number of reports of caffeinated beverage consumption was 4.7 ± 1.2 (range 3–8; IQR 4–6). Both models adjust for school day and demographic/household covariates: birth sex, race/ethnicity, body mass index (BMI) percentile, household income, primary caregiver’s education level, and whether the adolescent was living with two biological parents. Shaded light blue bands depict 95% confidence interval of caffeinated beverage consumption predicting each sleep measure. *b*, unstandardized beta (in hours); h, hours.

There were significant within-person associations between caffeinated beverage consumption and later sleep onset (*p* = 0.003), midpoint (*p* = 0.002), and offset (*p* = 0.011) (see Figure 2 and Table 3). On nights following consumption of ≥1 caffeinated beverage, adolescents’ sleep onset and midpoint were delayed by 0.28 h (17 min) and their sleep offset the next morning was delayed by 0.31 h (19 min), compared to nights following no caffeinated beverage consumption. There were no significant within-person associations of sleep duration (*p* = 0.968), sleep maintenance efficiency (*p* = 0.810), or subjective sleep quality (*p* = 0.703) and no significant between-person associations for caffeinated beverage consumption with any of these sleep measures (all *p* > 0.20).

We additionally examined whether the within-person effects of caffeinated beverage consumption on that night’s sleep varied depending on an adolescent’s average caffeinated beverage consumption. There were no significant interactions (all *p* > 0.17).

## 4. Discussion

The current study assessed bidirectional associations of caffeinated beverage consumption with multiple dimensions of sleep measured through actigraphy and subjective sleep quality in adolescents. Variability in sleep duration and midpoint across days was associated with higher average odds of an adolescent consuming ≥1 caffeinated beverage on a given day. Consuming ≥1 caffeinated beverage predicted later sleep timing that night compared to days on which the adolescent did not consume caffeine, but sleep timing did not predict next-day caffeinated beverage consumption. There were no within- or between-person associations of caffeinated beverage consumption with sleep duration or sleep quality (measured either through sleep efficiency or subjective ratings). These findings demonstrate that consuming caffeine may increase the likelihood that an adolescent shifts their sleep later and has more variable sleep duration and timing, potentially predisposing the adolescent to poorer psychological and physical health.

This study is the among the first to find that adolescents in a real-world study who consume caffeinated beverages more often are more likely to have variable sleep duration and timing. Specifically, for every extra *SD*-hour of variability in sleep duration and midpoint, adolescents were 17% and 21% more likely to consume at least one caffeinated beverage on average across monitoring days. We also measured sleep through objective measures, unlike previous studies that measured sleep through self-report [2,6,7,8,9,10,11,12,13,14,15,16,17,18,19,21,22,23,26,27,28,29,30,32,39]. Variable sleep schedules may contribute to daytime sleepiness due to circadian misalignment [60], which could lead to increased caffeinated beverage consumption to maintain alertness. Alternatively, later sleep timing, specifically on days when adolescents consume caffeine (as found in the current study), could drive greater variability across the week, particularly in those who consume more caffeine. Sleep variability has been associated with poor psychological and physical health, including mood instability [61] and increased odds of metabolic syndrome, greater adiposity, and poorer glycemic control [62]. More studies are needed to probe the link between caffeine consumption and sleep variability, particularly with objective measures of sleep.

We found that on days when adolescents consumed ≥1 caffeinated beverage, they fell asleep about 17 min later that night and woke up about 19 min later the next morning. The current study is one of the first to assess the link between caffeine consumption and objectively measured sleep timing in adolescents. The present findings suggest that adolescents may have more difficulty going to bed and rising early in time for morning activities such as school or extracurriculars after consuming caffeine. Similar to sleep variability, later sleep timing is a risk factor for poorer psychological [63] and metabolic health, including higher adiposity and BMI [64]. One study found that young adults with a 22 min earlier sleep onset and lower sleep variability following a light intervention reported higher positive affect compared to those with more delayed sleep onset [65], suggesting that even a minor advance in sleep timing and reduction of sleep variability may be associated with better psychological health. Furthermore, those with later sleep timing tend to perform poorly in the morning [66], which poses issues for adolescents who are expected to perform early due to school start times.

As both sleep onset and sleep offset were shifted later following caffeinated beverage consumption in the current study, there was no net effect on sleep duration. Previous studies examining the link between caffeine consumption and self-reported sleep duration in adolescents have been mixed [2,6,7,8,9,10,11,12,13,14,15,16,17,18,19,21,22,23], while two studies that measured sleep through actigraphy as in the current study found no cross-sectional association between caffeine consumption and sleep [21,22,23]. It is possible that self-reported and objectively measured sleep duration capture different constructs, and that adolescents who consume more caffeine perceive their sleep to be shorter without an actual difference in sleep duration.

We found no within- or between-person associations of caffeinated beverage consumption with either sleep efficiency or subjective sleep quality. These findings align with previous studies that found no association between caffeine consumption and wake after sleep onset [24,25] or self-reported [23] sleep quality. It should be noted that caffeine may have effects on nighttime sleep that are not captured by actigraphy, such as sleep onset latency or sleep architecture. For example, adolescents who consumed 80 mg caffeine 4 h before bedtime experienced unchanged total sleep time and sleep efficiency, yet they experienced increased non-rapid eye movement (REM) stage 1 (“light”) sleep and decreased non-REM stage 3 (“deep”) sleep [67], and sleep staging is not captured by actigraphy [33]. Such stage shifting may not translate into changes in the perception of sleep quality. Further research into bidirectional associations between caffeine consumption and both subjective and objective measures of sleep quality is warranted in adolescents.

We found that adolescents who consumed caffeinated beverages more often did not differ in average sleep duration, timing, efficiency, or subjective quality from other adolescents; that is, there were no between-person associations. It is possible that individuals develop a tolerance to caffeine consumption, such that caffeine no longer affects the sleep of habitual users. Tolerance to the effects of caffeine on sleep efficiency, for example, may develop after four days of caffeine consumption [68]. In addition, there is considerable interindividual variability in the effects of caffeine on sleep, with half-lives of caffeine consumption ranging from 2 to 10 h among individuals [69]. Adolescents in the current sample who were aware that caffeine did not affect their sleep may have been more likely to consume caffeinated beverages than more sensitive individuals. Tolerance to the effects of caffeine may explain why only the within-person association between caffeinated beverage consumption and sleep timing was significant in the current study, warranting further within-person research.

The current study has some limitations and certain strengths. Caffeinated beverage consumption was measured through daily self-report, which may not represent the adolescent’s routine consumption outside of the monitoring period. We also did not measure the timing of caffeinated beverage consumption, which is an important factor to consider given that caffeine consumed close to the nighttime sleep episode may have a stronger impact on that night’s sleep than caffeine consumed earlier in the day [70]. However, one study found that the impact of caffeine consumed 0, 3, or 6 h before bedtime on sleep was similar [36]. Furthermore, we did not account for caffeine content, which may vary among beverages such as coffee, tea, and energy drinks [71]. Future research may examine bidirectional associations of sleep and caffeine amount in adolescents. In addition, we cannot establish causal relationships within this observational study. A strength of the study is the large, diverse sample of adolescents across several areas of the United States. Other strengths of the current research are the objective measurement of multiple dimensions of sleep with actigraphy and the assessment of within-person effects, allowing for examination of temporal precedence.

## 5. Conclusions

The current study demonstrated that adolescents who consumed caffeinated beverages more frequently had greater sleep variability, and sleep was delayed on nights following daytime caffeinated beverage consumption. The findings suggest that reducing caffeinated beverage consumption in adolescents may assist in preventing negative consequences associated with sleep variability and delayed sleep timing, such as poor psychological [61,63] and metabolic [62,64] health.

## Figures and Tables

**Table 1:** Average descriptive statistics for analytical sample (*n* = 589).

Variable	*M* or %	*SD* or *n*
Demographic and household		
Age	15.39	0.52
Sex ^a^		
Female	53%	311
Male	47%	278
Race/ethnicity		
Black/African American	41%	240
Hispanic and/or Latino	25%	149
White/Caucasian	19%	112
Other, ^b^ mixed, or none	15%	88
Body mass index percentile ^c^	73.87	25.21
Annual household income (USD)	$64,906	$57,879
Primary caregiver’s highest education level		
Did not graduate high school	14%	85
High school graduate	18%	106
Completed some college	47%	276
College graduate	21%	122
Youth living arrangements		
Lives with 2 married/cohabiting biological parents	32%	187
Lives with <2 biological parents	68%	402
School attendance		
Attended school (proportion of days)	0.44	0.34
Nightly sleep measures		
Sleep duration (h)	7.79	1.08
Sleep onset (clock time)	0:28	1:44
Sleep midpoint (clock time)	4:21	1:42
Sleep offset (clock time)	8:20	1:47
Sleep maintenance efficiency (%)	90.70	3.43
Subjective sleep quality ^d^	2.36	0.50
Sleep variability measures ^e^		
Variability (*SD*) of sleep duration (h)	1.57	0.80
Variability (*SD*) of sleep onset (h)	1.30	0.74
Variability (*SD*) of sleep midpoint (h)	1.22	0.65
Variability (*SD*) of sleep offset (h)	1.56	0.90
SRI ^f^	48.35	13.38
Social jetlag (h) ^g^	1.79	1.15
Dietary intake		
Consumed ≥1 cup caffeinated beverage (proportion of days) ^h^	0.61	0.34
Consumed ≥1 caffeinated beverage 0–24% of the days	19%	112
Consumed ≥1 caffeinated beverage 25–49% of the days	15%	88
Consumed ≥1 caffeinated beverage 50–74% of the days	22%	129
Consumed ≥1 caffeinated beverage 75–100% of the days	44%	260

Notes. The mean number of actigraphy recordings per adolescent was 5.6 ± 1.4 (range 3–10; IQR 5–7) and the mean number of reports of caffeinated beverage consumption was 5.5 ± 1.4 (range 3–9; IQR 4–7). ^a^ Data collected at birth. ^b^ Other category includes Asian, Central American/Caribbean, Native American/Alaska Native, and/or Native Hawaiian/Pacific Islander. ^c^ Calculated based on 2000 Centers for Disease Control and Prevention (CDC) growth charts, matched for age and sex [53]. ^d^ Ranges from 0 (very bad)–3 (very good). ^e^ Higher value means greater variability, except the reverse for the sleep regularity index. ^f^ Calculated based on formula from Phillips et al. [51]; ranges from 0 (low)–100 (high). ^g^ Calculated based on formula from Wittmann et al. [52]. *n* = 370 (adolescent included only if provided at least one school night and one free night of actigraphy). ^h^ Includes coffee or tea, caffeinated soda, and energy drinks. H, hours; *M*, mean; *n*, number; *SD*, standard deviation; SRI, sleep regularity index; USD, United States dollar.

**Table 2:** Between-person associations of sleep variability per youth across monitoring days with average odds of caffeinated beverage consumption (*n* = 589).

Model Predictor	OR	95%CI OR	
Sleep duration (*SD*, h)	1.21 *	1.01	1.45
Sleep onset (*SD*, h)	1.19 ^†^	0.97	1.46
Sleep midpoint (*SD*, h)	1.27 *	1.01	1.59
Sleep offset (*SD*, h)	1.17 ^†^	1.00	1.38
SRI ^a^	0.99	0.98	1.00
Social jetlag (h) ^b^	1.07	0.91	1.25

Notes. Each row represents a separate multilevel model that adjusts for mean sleep duration (linear and quadratic, sleep duration × sleep duration) and demographic/household covariates: birth sex, race/ethnicity, household income, and primary caregiver’s highest education level. Caffeinated beverage consumption (the outcome) includes coffee or tea, caffeinated soda, and energy drinks and was coded as 0 = none; 1 = at least one 8 oz beverage that day. The between-person effect for sleep variability measures is represented by *SD* or SRI [51] (across all time points) or social jetlag (average midpoint on free nights−average sleep midpoint on school nights [52]) The mean number of valid actigraphy nights per youth included in present analyses was 5.6 ± 1.4 (range 3–10; IQR 5–7) and the mean number of reports of caffeinated beverage consumption was 5.5 ± 1.4 (range 3–9; IQR 4–7). Higher value means greater variability, except the reverse for the SRI. ^a^ Calculated based on formula from Phillips et al. [51]; ranges from 0 (low)–100 (high). ^b^ Calculated based on formula from Wittmann et al. [52]. *n* = 370 (adolescent included only if provided one school night and one free night of actigraphy). ^†^ *p* < 0.10, * *p* < 0.05, two-tailed. CI, confidence interval; h, hours; OR, odds ratio; *SD*, standard deviation; SRI, sleep regularity index.

**Table 3:** Caffeinated beverage consumption predicting sleep within and between adolescents (*n* = 458).

Model Outcome	Within-Person			Between-Person		
	*b*	95% CI		*b*	95% CI	
Sleep duration (h)	<0.01	−0.22	0.23	0.04	−0.24	0.32
Sleep onset (h)	0.28 **	0.10	0.47	−0.08	−0.46	0.30
Sleep midpoint (h)	0.28 **	0.11	0.46	−0.06	−0.41	0.30
Sleep offset (h)	0.31 *	0.07	0.55	−0.05	−0.41	0.32
Sleep maintenance efficiency (%)	−0.05	−0.45	0.35	−0.13	−0.98	0.72
Subjective sleep quality ^a^	0.01	−0.05	0.08	−0.07	−0.20	0.06

Notes. Each row represents a separate multilevel model that adjusts for school attendance and demographic/household covariates: birth sex, race/ethnicity, body mass index (BMI) percentile, household income, primary caregiver’s education level, and whether the adolescent was living with two biological parents. Caffeinated beverage consumption (predictor) includes coffee or tea, caffeinated soda, and energy drinks and was coded as 0 = none; 1 = at least one 8 oz beverage that day. Sleep timing measures (onset, midpoint, and offset) were centered around midnight (0:00). The mean number of valid actigraphy nights per youth included in present analyses was 5.2 ± 1.1 (range 3–9; IQR 4–6) and the mean number of reports of caffeinated beverage consumption was 4.7 ± 1.2 (range 3–8; IQR 4–6). ^a^ Ranges from 0 (very bad)–3 (very good). * *p* < 0.05, ** *p* < 0.01, two-tailed. *b*, unstandardized beta coefficient; CI, confidence interval; h, hours.

## Data Availability

Survey data from the Fragile Families and Child Wellbeing study (https://fragilefamilies.princeton.edu/documentation), accessed on 22 March 2017. are publicly available from Princeton University’s Office of Population Research (OPR) data archive: https://opr.princeton.edu/archive/restricted/Default.aspx (accessed on 22 March 2017). The sleep actigraphy and daily diary datasets generated and analyzed during the current study (accessed on 11 August 2020) are not publicly available yet but will be available through an application process at the above link.

## Supplementary Materials

The following are available online at https://www.mdpi.com/article/10.3390/nu14010031/s1, Figure S1: Participant flow chart for sample included in analyses where sleep predicts caffeinated beverage consumption and sleep variability analyses (*n* = 589 except for social jetlag, *n* = 370); Figure S2: Participant flow chart for sample included in analyses where caffeinated beverage consumption predicts sleep (*n* = 458). Table S1: STROBE-nut: An extension of the STROBE statement for nutritional epidemiology; Table S2: Intraindividual consistency of repeated-measures variables.
